# Relationship between Organophosphate and Pyrethroid Insecticides in Blood and Their Metabolites in Urine: A Pilot Study

**DOI:** 10.3390/ijerph17010034

**Published:** 2019-12-18

**Authors:** Sailent Rizki Sari Simaremare, Chien-Che Hung, Chia-Jung Hsieh, Lih-Ming Yiin

**Affiliations:** 1 Institute of Medical Sciences, Tzu Chi University, 701, Sec. 3, Zhongyang Road, Hualien City 97004, Taiwan; 103324121@gms.tcu.edu.tw (S.R.S.S.); gavink23@gmail.com (C.-C.H.); 2 Department of Public Health, Tzu Chi University, 701, Sec. 3, Zhongyang Road, Hualien City 97004, Taiwan; cjhsieh@mail.tcu.edu.tw

**Keywords:** blood, insecticide, metabolite, organophosphate, pyrethroid, urine

## Abstract

We conducted a pilot study to examine the relationship between organophosphate (OP) and pyrethroid (PYR) insecticides in blood and their metabolites in urine. A total of 30 pregnant women were enrolled in the study, and blood and urine was sampled from each subject during a regular clinic visit. Two OP and nine PYR insecticides were selected for blood sample analysis, while six OP and five PYR metabolites were analyzed for urine specimens. Both types of samples were processed and analyzed on gas chromatography-mass spectrometry. For OPs in blood, chlorpyrifos had a higher mean concentration (73.33 µg/L) than terbufos. For PYRs in blood, cypermethrin and imiprothrin were the most frequently detected species with the highest mean concentrations (151.25 and 141.25 µg/L). The concentrations of PYRs appeared to be higher than that of OPs, and the most frequently detected PYRs were commonly used in domestic products, suggesting that the exposure could mostly originate from use of domestic insecticides. The correlation between insecticides in blood and their metabolites in urine was significantly high (*r* = 0.795 for OPs and 0.882 for PYRs, *p* < 0.001), indicating routine exposure at a steady state. Residents should be cautious with domestic use of insecticide products to lower their exposure.

## 1. Introduction

Organophosphates (OPs) and pyrethroids (PYRs) are among the most commonly used insecticides globally. Despite the declining trend in usage in general since 2007, the projected amounts of OPs and PYRs are the majority among the insecticide groups [1]. OPs and PYRs have been widely used in agriculture for crop protection, and for non-agricultural purposes, such as control of vector-borne diseases, turf and ornamental protection, livestock and veterinary applications, and residential pest control; they are even applied in personal care products such as shampoo and mosquito-repellent perfume [2,3].

OPs act as acetylcholinesterase (AChE) inhibitors by binding to the serine residue in the active site of AChE and preventing the normal metabolism of acetylcholine. Wildlife and humans could have the same effect when exposed to OPs, because this reaction does not only apply to insects [4]. The nerve action potential primarily is mediated through the transient increase in the sodium permeability of the nerve membrane [5], and PYRs exhibit neurotoxic effects by modulating the sodium channel voltages. Although PYRs are more selective for their target species than OPs, administration of a highly effective dose can produce neurotoxicity in non-target species, including mammals [6]. There have been growing studies reporting chronic exposure to OPs and PYRs linked to adverse health outcomes such as respiratory diseases [7], neurological symptoms [8], potential neurodevelopmental disorder [9,10,11], hormonal and reproductive disruption [12], chronic diseases [13] and risk of cancers [14,15,16]. Needless to say, the acute effects of poisoning by these insecticides are well known [17]. Thus, the widespread use and toxicity of OPs and PYRs poses a major risk to the general population, especially susceptible groups, such as children and pregnant women.

Environmental exposure to OPs and PYRs could occur via ingestion primarily by consumption of food contaminated with pesticide residues [18], and via inhalation or ingestion of contaminated household dust after indoor application of insecticides [19]. After absorption by the organs, these insecticides appear in the blood circulatory system, and thus the blood specimens taken from the body are considered representative of the instant exposure. The absorbed insecticides then undergo the metabolic process in two phases. In phase I reactions the metabolic enzymes change the insecticide compounds to more water soluble products than the originals via reduction, oxidation or hydrolysis, whereas in phase II reactions, the conjugation with hydrophilic molecules occurs to increase the water solubility for excretion in urine [20].

Most of OPs are metabolized in the human body to dialkyl phosphate (DAP) metabolites. Commonly six urinary dialkyl phosphate metabolites of OPs are measured for biomonitoring, including dimethylphosphate (DMP), dimethylthiophosphate (DMTP), dimethyldithiophosphate (DMDTP), diethylphosphate (DEP), diethylthiophosphate (DETP), and diethyldithiophosphate (DEDTP) [21]. PYRs are commonly metabolized in the body via ester cleavage of the original compounds to trans-chrysanthemumdicarboxylic acid (*trans*-CDCA), *cis-* and *trans*-3-(2,2-dichlorovinyl)-2,2-dimethylcyclopropanecarboxylic acid (*cis*-/*trans*-DCCA), *cis*-3-(2,2-dibromovinyl)-2,2-dimethylcyclopropanecarboxylic acid (*cis*-DBCA), 3-phenoxybenzoic acid (3-PBA), and 4-fluoro-3-phenoxybenzoic acid (F-PBA) [22]. The commonly used OPs and PYRs are confirmed to be metabolized in the human body quickly with half-lives of hours to a few days, evidenced by a number of animal and human studies [23,24,25,26,27,28,29,30,31,32]; therefore, these metabolites are usually excreted in urine within days, reflecting the exposure of the latest few days. Determination of the concentrations of these insecticides in blood or their respective metabolites in urine is an effective method to assess human exposure to insecticides [19]. Monitoring of OPs and PYRs in blood can be done through direct measurement of parent compounds, which accurately reflect the absorbed dose or potential dose, whereas monitoring of OPs and PYRs in urine is easy and non-invasive by measuring their representative metabolites [33]. Despite the advantage possessed by blood specimens, most researchers tend to collect urine, instead of blood, for biomonitoring probably because it is an easy and non-invasive procedure. There are a few studies focusing on blood sample analysis for insecticides [34,35,36]; the results, however, are non-comparable to that of studies using urine sampling and vice versa. Little is known about the relation between insecticide data derived from blood and urine, although it is presumably present.

We conducted a pilot study to examine the relationship between the contents of selected insecticides (OPs or PYRs) in blood and that of their representative metabolites in urine (Table 1). The study aimed to evaluate the insecticide concentrations in the form of parent compounds in blood and of their metabolites in urine, and to find the association between these two types of biomonitoring data. Based on an assay method previously developed by our research team [37], we were able to analyze the human specimens accurately and precisely. The result presented herein expects to establish a bridge across the gap between the exposure results generated from blood and urine sampling, and to help understand the domain of human exposure to insecticides better.

## 2. Materials and Methods

### 2.1. Study Subjects and Sample Collection

The study subjects were pregnant women in their second to the third trimester, who were randomly selected from participants of a research project of maternal exposure to insecticides. The study consent and protocols were reviewed and approved by the Ethical Committee of Tzu Chi General Hospital/University (No. IRB102–71, approved on 8 October 2015). Blood and urine samples were collected from each of the 30 subjects during a check-up visit. The blood sampling method was performed by intravenous sampling with an empty needle, and 15 mL of blood was drawn into a blood collection tube without anticoagulant, which was transferred and stored in an ultra-low temperature freezer at −80 °C for later processing and analysis.

Urine was collected in a temporary storage container, and 50 mL was taken and stored in a sealed bottle, temporarily stored in a household refrigerator at 4 °C during the visit and transferred to a −80 °C ultra-low temperature freezer prior to analysis. Each urine sample was tested with 100 μL of the abcam creatinine assay kit prior to extraction to confirm that the sample urine was normally metabolized for analysis.

### 2.2. Chemicals and Materials

**Figure 1:**
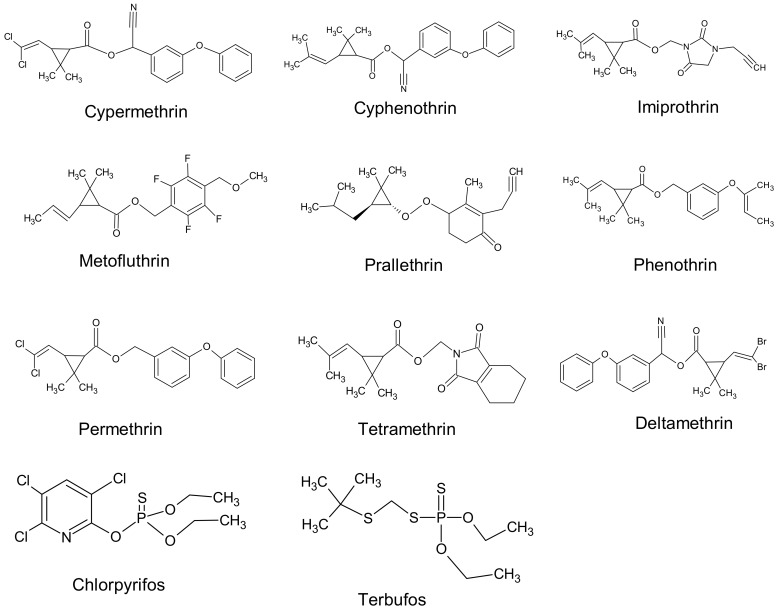
Chemical structures of organophosphate (OP) and pyrethroid (PYR) insecticides measured in the study.

**Figure 2:**
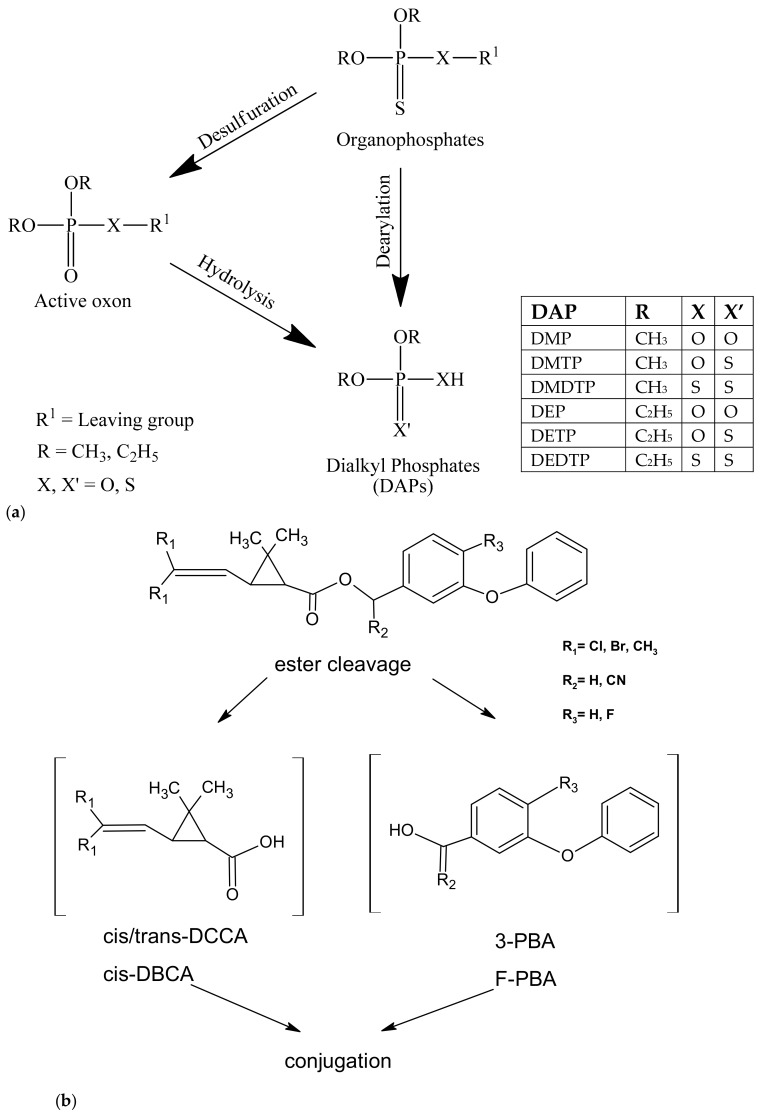
Metabolisms of (**a**) OPs and (**b**) PYRs and structures of metabolites measured in the study (adapted from [37]).

We analyzed the most commonly used insecticides in Taiwan, two OPs and nine PYRs (Figure 1), and 11 metabolites, six originating from OPs and five from PYRs (Figure 2). These compounds, with other chemicals and solvents, were all commercially available. Analytical grade acetonitrile, analytical grade *n*-hexane, analytical grade methanol, ammonia solution (2.0 M in ethanol), hydrogen chloride (99.5%), butyl chloride (≥99.8%), *trans*-CDCA (95.5%), cyphenothrin (98.4%), DEDTP (95%), ethion (analytical standard, used as internal standard for blood analysis), *N*-*tert*-butyldimethylsilyl-*N*-methyltrifluoroacetamide (MTBSTFA, 97.5%, used for derivatization), and terbufos (95%) were purchased from Sigma-Aldrich (Saint Louis, MO, USA). Chlorpyrifos (99.5%), prallethrin (99%), and tetramethirn (99%) were purchased from Chem Service (West Chester, PA, USA). Prallethrin (99%), tetramethirn (99%), permethirn (99.7%), cypermethirn (98.4%), deltamethirn (99.5%), and DETP (99.5%) were purchased from TCI (Tokyo, Japan). *Cis/trans*-DCCA (97.5%) and DMTP (95.5%) were purchased from TRC (Toronto, ON, Canada). DMDTP (99.5%) and 3-PBA (98%), and 2-PBA (98%, the internal standard for PYR metabolite measurements) were obtained from Alfa Aesar (Lancashire, UK). DEP (99.5%) and DMP (98%) were obtained from ACROS (Geel, Belgium). *cis*-DBCA 98.9% was purchased from Bayer AG (Leverkusen, Germany), and blank Winstar rat blood (used for preparation of standards) was purchased from Merck (Kenilworth, NJ, USA).

### 2.3. Blood Analysis

#### 2.3.1. Pre-Analysis Treatment

The blood samples were analyzed for the 11 targeted insecticides, which were commonly used for agriculture and/or environmental sanitation. Having been defrosted, the whole blood samples were ultrasonically oscillated to ensure homogenization and thorough mixing, and dispensed into several 1.5 mL brown sample vials, which were stored at −20 °C prior to sample processing. A 0.5 mL blood sample was required for a single analysis. Each 0.5 mL blood sample was mixed with a 20 μL ethion solution of 10 mg/L in methanol as an internal standard solution and 1.75 mL of 4.5% ammonia solution in a 15 mL brown glass bottle, which was then vortexed for 20 s for homogenization. Ten milliliters of butyl chloride were added to the bottle, which was oscillated at 20–25 °C for 10 min. The sample was then centrifuged in a low temperature centrifuge at 6000× *g* for 10 min at 4 °C. The supernatant was transferred to a 1.5 mL brown sample vial and concentrated using a programmable automatic vacuum concentrator (miVac Duo concentrator, SP Scientific, Ipswich, Suffolk, UK) until dryness. The sample was reconstituted with the addition of 100 μL of methanol, and the vial was immediately covered, sealed, and stored in a freezer at −20 °C prior to analysis.

#### 2.3.2. Analysis on Gas Chromatography Mass Spectrometry (GCMS)

The analytical instrument used in this study was Agilent 6890 GC/5973 MS (Agilent, Santa Clara, CA, USA). An RTX-25 column (25 m × 0.25 mm × 0.25 μm, Restek, Bellefonte, PA, USA) was used with the temperature starting at 70 °C, then increasing to 160 °C at 20 °C/min, to 300 °C at 10 °C/min, and held for 8.5 min. Interface temperature was set at 280 °C, inlet temperature was at 270 °C, and ion source was at 230 °C. One microliter of a sample was injected into the system with a split ratio of 1/50, and the flow rate was set at 1 mL/min with the mobile phase of high purity helium (5n5, purity >99.995%). The mass spectrometry scanning method was selected ion monitoring (SIM), and a quantitative ion was matched with one or two confirmation ions. The method used was effective and reliable with a limits of detection (LODs) range from 0.025–0.1 μg/L, and limits of quantification (LOQs) from 0.1–0.3 μg/L for the 11 insecticide analytes.

### 2.4. Analysis of Urinary Metabolites

Each urine sample was dispensed into several 5 mL Teflon-capped brown glass bottles and stored at −20 °C prior to processing and analysis. Metabolites of OPs and PYRs were processed and analyzed following the method developed previously [37]. Since most of the metabolites underwent Phase II metabolism, de-conjugation had to be conducted via acid hydrolysis. Fifty percent of analytical grade hydrochloric acid was used for acid hydrolysis, and 2-PBA was added as internal standard. Sample extraction was conducted using *n*-hexane, and the extract was concentrated to dryness in the miVac Duo concentrator. The sample was reconstituted with 100 μL of acetonitrile, and MTBSTFA was added for derivatization, which lowered the polarity for the following GCMS analysis.

The same GCMS system was used for urine analysis with the mobile phase being high purity helium, but with a different RTX-35 column (35 m × 0.25 mm × 0.25 μm, Restek, Bellefonte, PA, USA). The scanning method was also SIM with different settings (e.g., temperatures) from that for blood analysis. The LODs ranged from 0.025–0.1 μg/L, and LOQs ranged from 0.1–0.3 μg/L for the 11 metabolite analytes. Details of process and analysis were described previously [37].

### 2.5. Data Management and Statistical Analysis

Descriptive statistics was performed for each insecticide or metabolite, and a mean value was calculated from all 30 individual concentrations even including non-detects (zero for those under LOD). For correlation analysis between blood and urine data, all concentrations in blood or urine (μg/L) were converted to molar concentrations (nmol/L), which could be summarized within the same category (OP or PYR). Thus, each of the 30 subjects had a pair of a concentration of total insecticides in blood and a concentration of total metabolites in urine. Pearson correlation analysis on the log-transformed values was performed to test the relationship between OP or PYR insecticides in blood and the respective metabolites in urine. Metabolite concentrations in urine (μg/L) were used rather than creatinine-adjusted concentrations, because all urine samples were confirmed to be metabolized normally at prior kit tests, suggesting that such an adjustment might not be necessary. Statistical analysis was performed using SPSS statistical software package version 23.0 (SPSS Inc., Chicago, IL, USA), and descriptive statistics were computed in Microsoft Excel^®^ (Microsoft, Redmond, WA, USA).

## 3. Results

### 3.1. Detection of OPs and PYRs in Human Blood

The analytic results of OPs and PYRs in blood are shown in Table 2. For OPs, terbufos was the more frequently detected species with a detection rate of 53.3% than chlorpyrifos (36.7%). Although detected less frequently than terbufos, chlorpyrifos was almost twice as high in mean concentration (73.33 µg/L) than terbufos (48.84 µg/L). As for PYRs, imiprothrin (80.0%) was detected the most frequently followed by cypermethrin (66.7%) and cyphenothrin (66.7%), and the rest all went under 50% in detection rate. Although cyphenothrin was among the most frequently detected PYR, the concentration (56.51 µg/L) was far lower than those of imiprothrin (141.25 µg/L), cypermethrin (151.25 µg/L), and metofluthrin (101.25 µg/L). Tetramethrin was the least frequently detected species (13.3%) with the lowest mean concentration (33.42 µg/L), followed by deltamethrin with a detection rate of 33.3% and a mean concentration of 39.32 µg/L. Despite a detection rate less than 50%, prallethrin had the highest in mean concentration (161.25 µg/L), suggesting high doses of occasional exposures to prallethrin.

### 3.2. Detection of OPs and PYRs Metabolites in Urine

Data of the OP and PYR metabolites in urine are given in Table 3. For OP metabolites, the detection rates of urinary metabolites were less than 40%, with detection of methyl metabolites (19 detects in total) being fairly higher than that of ethyl metabolites (17 in total). DMTP was the most frequently detected (33.3%) with a relatively low mean concentration (3.91 µg/L), followed by DEDTP (20.0%, 3.25 µg/L), and DEP (20.0%, 2.05 µg/L). DMP and DETP had the highest mean concentrations (8.02 and 7.53 µg/L, respectively), albeit with the same low detection rate (16.7%). For PYR metabolites, *cis*-DBCA and *trans*-DCCA were the most frequently detected (both of 73.3%), followed by *cis*-DCCA (66.7%), *trans*-CDCA (56.7%), and 3-PBA (36.7%). In addition to the most frequently detected, *trans*-DCCA was found to have the highest mean concentration (19.25 µg/L) among the PYR metabolites, followed by *cis*-DBCA (17.25 µg/L), *trans*-CDCA (13.36 µg/L), 3-PBA (8.85 µg/L), and *cis*-DCCA (4.93 µg/L). It appears that exposure to PYRs, shown by the blood or urinary biomarkers, was higher than that to OPs.

### 3.3. Correlation between Insecticides in Blood and Metabolites in Urine

**Figure 3:**
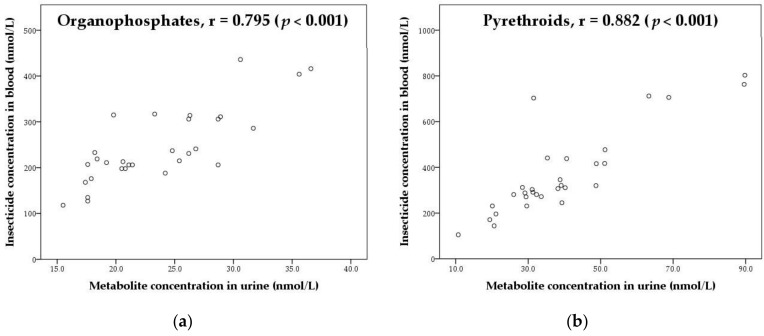
Correlation between insecticides in blood and metabolites in urine: (**a**) OPs and (**b**) PYRs.

Molar concentrations of total insecticides in blood and that of total metabolites in urine from the 30 subjects are plotted in Figure 3. The correlation for either insecticide was significantly high (*r* = 0.795 and 0.882 for OPs and PYRs, respectively), indicating that both types of biomonitoring data, used as tools of exposure assessment, would be indicative with the generation of consistent results with each other. The summarized molar concentrations in blood and urine, however, were approximately ten-fold different, suggesting that only certain portions of insecticides in blood would be metabolized to the selected metabolites that were excreted in urine.

## 4. Discussion

In Taiwan, chlorpyrifos is used for both agriculture and environmental sanitation, while terbufos is only used for agricultural purposes [38]. The high detection rate of terbufos suggests that these subjects may have been exposed to insecticides originating from nearby agricultural areas environmentally, or simply via dietary digestion of contaminated vegetables. The former scenario is improbable, because no environmental terbufos was detected from indoor or outdoor dust in these homes, as shown by our previous work [38]; thus, the latter (dietary digestion) was the most likely answer. This inference is reasonable because agricultural produce is regulated to contain no or limited insecticide residue, which would usually build up low levels of insecticides in the human body; in addition, dietary ingestion is a constant exposure route, and thus the detection rate of insecticides could be within a moderate range. Although chlorpyrifos had a lower detection rate than that of terbufos, the mean concentration was almost twice as high, suggesting the occasional use of household insecticides containing chlopyrifos. Despite the relatively low detection rate of chlorpyrifos (36.7%), it was higher than those reported by several U.S. studies working in urban areas [39,40]. The lower detection rates shown in the U.S. studies are rational because the ban of chlorpyrifos in domestic use became effective in 2000. A similar comparison result was made in our previous work [38], demonstrating relatively low levels of chlorpyrifos in the dust of U.S. houses compared to our Taiwanese data. As chlorpyrifos and terbufos have been banned for domestic use respectively in the U.S. and in China and European Union [41], exposure to OPs should be of concern in Taiwan.

The six DAP metabolites were detected from less than 40% of the subjects with methyl metabolites being more frequently detected than ethyl metabolites, and this result was consistent with findings from other studies [42,43]. As mentioned previously, chlorpyrifos and terbufos are the major OPs used in the market, and the main metabolites should be DEP and DETP, according to the structures of parent compounds (with ethyl functional groups). The finding showing the fairly higher detection rates for methyl metabolites suggests that methyl chlorpyrifos (or terbufos) could be the major ingredient of insecticide products. Since insecticide products are usually mixtures of similar compounds (e.g., methyl and ethyl chlorpyrifos) and metabolites are not exclusively from certain parent compounds (e.g., DEP possibly from chlorpyrifos, terbufos, or other OPs), the relationships between parent compounds and metabolites are better to evaluate by type (e.g., OP, PYR) than to check individual insecticides.

In this study, imiprothrin (80.0%) and cypermethrin (66.7%) were the most frequently detected PYRs, and also among those with the highest mean concentrations. Others among the top mean concentrations were metofluthrin and prallethrin, albeit with relatively low detection rates. These four PYRs matched the most common ingredients of insecticide products available in the Taiwanese market, according to a survey conducted by our research team. This result of PYRs suggests that the subjects should have been exposed to insecticides environmentally more than via the dietary ingestion route because all but cypermethrin are used for environmental sanitation only. The finding of a high detection rate and high mean concentration of cypermethrin is consistent with our previous work indicating cypermethrin as the most frequently detected PYR in house dust [38]. Prallethrin is commonly known for the active ingredient of electric mosquito incense, which customers usually use overnight for mosquito prevention. Thus, occasional use of the product could lead to a low detection rate but a high mean concentration, as shown in Table 2.

For PYR metabolites, *cis*-DBCA (73.3%) and *trans*-DCCA (69.8%) were the most frequently detected metabolites, followed by *cis*-DCCA (66.7%), *trans*-CDCA (56.7%), and 3-PBA (36.7%). The high detection rates of *cis*-/*trans*-DCCA with a high total of mean concentrations in this study indicate extensive use of the parent PYRs, such as cypermethrin, permethrin, and cyflutrhin; the concentrations of cypermethrin and permethrin found in the blood specimens could be evidence. The highest detection rate of *cis*-DBCA with a relatively high mean concentration (17.25 µg/L) indicates frequent exposure to deltamethrin because *cis*-DBCA is considered an exclusive metabolite of deltamethrin [44]. In contrast, we only detected deltamethrin from a third of the blood samples, indicating that the results from blood and urine were not consistent. A possible explanation is that blood samples indicate the instant exposure and urine samples reflect exposure of the recent days (after a certain period of time for metabolism), and both exposures may not be related at certain points of time. The lowest detection rate of 3-PBA found in this study was in contrast with the studies [45]. Although 3-PBA is generated from metabolisms of various PYRs (e.g., cypermethrin, deltamethrin, permethrin), the metabolisms starting with different parent compounds could have 3-PBA in different yields; in addition, 3-PBA has been reported to undergo further oxidation to 3-(4′-hydroxyphenoxy) benzoic acid [30]. Therefore, the low detection rate of 3-PBA was not of surprise.

*Trans*-DCCA (19.25 µg/L) was found to have the highest mean concentration among the PYR metabolites, whereas its isomer, *cis*-DCCA (4.93 µg/L), had the lowest. A study testing urinary metabolites of a single dose of *cis*-/*trans*-cypermethrin (1:1) found that excretion of metabolites from *trans*-isomer was more than that from *cis*-isomer [46]. This is because *trans*-isomers tend to be hydrolyzed more effectively than *cis*-isomers [47]. Thus, our finding of more *trans*-DCCA than *cis*-DCCA is consistent with the outcomes of these previous studies. We did not know the composition of *cis*-/*trans*-PYRs to which the subjects were exposed, but it is likely that *trans*-PYRs in a larger portion of insecticide products were one of the reasons as well. Overall, the subjects were exposed to PYRs more than OPs shown by either the blood or urinary biomonitoring data. This finding suggests that environmental exposure other than dietary ingestion may play a major role, because the detected PYRs with high mean concentrations are the common ingredients of domestic products.

Following the principles of physiologically based pharmacokinetics, insecticides in blood are eventually transformed to hydrophilic metabolites for urinary excretion. A bi-exponential pattern of elimination, a rapid phase followed by a slow phase, fits the metabolisms with half-lives being several hours to a few days [23,24,25,26,27,28,29,30,31,32]. Constant intake and elimination of insecticides (e.g., OPs, PYRs) would reach a steady-state equilibrium in the body [48], in which the levels of insecticides in the blood or that of metabolites in urine remain stable. The high correlation result for OPs or PYRs in this study actually reflects the stable status, which indicates that the subjects could be exposed to either insecticide on a daily basis. As a result, either blood or urine sampling could be used for biomonitoring, albeit with different indicative information (i.e., distinguishable insecticides, groups of metabolites of types).

There are several limitations in the study. Firstly, this was a pilot study with a small sample size (*n* = 30) and no duplication of sampling; under such a circumstance, we could not conduct a multivariate analysis (e.g., principal component analysis) or an adjustment for covariates (e.g., age, blood pressure), which requires a large sample size. Fortunately, the result came up with significance, indicating the relationship between blood and urine data was apparent. Secondly, insecticides and metabolites selected for analysis were limited, and there must have been missing insecticides or metabolites in the analysis; it appeared that the selected analytes were sufficient to establish the blood–urine relationship. With more insecticides and metabolites included in the analysis, it is believed that the relationship could be better. Thirdly, subject recruitment was conducted by convenience sampling, which might have resulted in participation bias; thus, the exposure data derived from this study might not be appropriate to represent that of the population. Finally, because the result of this study was not linked with the questionnaire data, the sources of insecticide exposure could only be inferred from information that blood samples revealed. The inference might not cover all sources of insecticides, but what it covered should be as close as the truth.

## 5. Conclusions

The pregnant subjects in this study were widely exposed to OP and PYR insecticides. The majority of exposure was considered to originate from frequent use of domestic insecticides for insect control, and the minority was from contaminated food via dietary ingestion. The high correlation between OP or PYR insecticides in blood and their metabolites in urine indicates routine exposure to those insecticides at a steady state. Residents, especially pregnant women, should be cautious with domestic use of insecticide products to lower their exposure. As OPs are banned or phased out by a number of countries due to their health effects, the use of OPs in agricultural and domestic products in Taiwan is of concern.

## Figures and Tables

**Table 1:** The main urinary metabolites of common organophosphate (OP) and pyrethroid (PYR) insecticides (modified from [37]).

Metabolite	Parent Insecticide	Classification
DMP	Azinphos methyl, Chlorpyrifos methyl, Dichlorvos (DDVP), Dicrotophos, Dimethoate, Fenitrothion, Fenthion, Methyl parathion, Oxydemeton-methyl, Phosmet, Pirimiphos-methy, Temephos, Naled, Tetrachlorviphos, Trichlorfon	OP
DMTP	Azinphos methyl, Chlorpyrifos methyl, Dimethoate, Isazaphos-methyl, Fenitrothion, Fenthion, Methyl parathion, Oxydemeton-methyl, Phosmet, Pirimiphos-methy, Temephos,	OP
DMDTP	Azinphos methyl, Dimethoate, Malathion, Phosmet,	OP
DEP	Chlorethoxyphos, Chlorpyrifos, Coumaphos, Diazinon, Disulfoton, Ethion, Malathion, Parathion, Phorate, Sulfotepp, Terbufos,	OP
DETP	Chlorethoxyphos, Chlorpyrifos, Coumaphos, Diazinon, Disulfoton, Ethion, Parathion, Phorate, Sulfotepp, Terbufos,	OP
DEDTP	Disulfoton, Ethion, Phorate, Terbufos,	OP
3-PBA	Cyhalothrin, Cypermethrin, Deltamethrin, Ethonfenprox, Esfenvalerate, Fenpropathrin, Permethrin, Phenothrin	PYR
*Cis/trans*-DCCA	Cypermethrin, Cyfluthrin, Permethrin	PYR
*Cis*-DBCA	Deltamethrin	PYR
*Trans*-CDCA	Allethrin, Imiprothrin, Phenothrin, Prallethrin, Resmethrin, Tetramethrin	PYR
FPBA	Cyfluthrin	PYR

Abbreviations: DMP, dimethylphosphate; DMTP, dimethyl thiophosphate; DMDTP, dimethyl dithiophosphate; DEP: diethyl phosphate; DETP, diethyl dithiophosphate; DEDTP, diethyl dithiophosphate; 3-PBA, 3-Phenoxybenzoic Acid; *Cis*/*trans*-DCCA, *cis*- and *trans*-3-(2,2-Dichlorovinyl)-2,2-dimethylcyclopropanecarboxylic acid; *Cis*-DBCA, *cis*-3-(2,2-Dibromovinyl)-2,2-dimethylcyclopropanecarboxylic acid; *Trans*-CDCA, trans-chrysanthemumdicarboxylic acid; FPBA, 4-fluoro-3-phenoxybenzoic acid.

**Table 2:** Concentrations of OP and PYR insecticides in blood (*n* = 30).

Classification	Insecticide	Detected *n* (%)	Mean ± SD (µg/L)	Median (µg/L)	Maximum (µg/L)
OP	Chlorpyrifos	11 (36.7)	73.33 ± 11.17	41.35	117.69
	Terbufos	16 (53.3)	48.84 ± 10.21	19.21	65.25
PYR	Cypermethrin	20 (66.7)	151.25 ± 4.45	79.21	153.25
	Cyphenothrin	20 (66.7)	56.51 ± 8.58	26.53	67.73
	Deltamethrin	10 (33.3)	39.32 ± 3.35	33.61	58.32
	Imiprothrin	24 (80.0)	141.25 ± 3.35	32.33	192.21
	Metofluthrin	10 (33.3)	101.25 ± 8.33	55.21	136.32
	Permethrin	5 (16.7)	94.33 ± 2.25	70.25	165.32
	Phenothrin	8 (26.7)	65.32 ± 8.77	41.10	88.36
	Prallethrin	11 (36.7)	161.25 ± 3.28	88.61	191.25
	Tetramethrin	4 (13.3)	33.42 ± 6.98	8.85	75.86

Abbreviation: OP, organophosphate; PYR, pyrethroid; SD, standard deviation.

**Table 3:** Concentrations of urinary metabolites of OP and PYR (*n* = 30).

Pesticides	Metabolites	Detected *n* (%)	Mean ± SD (µg/L)	Median (µg/L)	Maximum (µg/L)
OP	DEP	6 (20.0)	2.05 ± 2.11	1.96	7.17
	DETP	5 (16.7)	7.53 ± 1.71	4.47	8.23
	DEDTP	6 (20.0)	3.25 ± 1.16	1.33	7.11
	DMP	5 (16.7)	8.02 ± 4.49	4.23	8.33
	DMTP	10 (33.3)	3.91 ± 0.83	1.19	4.43
	DMDTP	4 (13.3)	4.49 ± 0.77	3.38	6.94
PYR	*cis*-DCCA	20 (66.7)	4.93 ± 1.81	2.71	71.3
	*trans*-DCCA	22 (73.3)	19.25 ± 3.38	11.31	34.6
	*cis*-DBCA	22 (73.3)	17.25 ± 3.19	5.51	22.5
	*trans*-CDCA	17 (56.7)	13.36 ± 2.88	6.33	18.56
	3-PBA	11 (36.7)	8.85 ± 1.17	8.52	20.91

Abbreviation: OP, organophosphate; PYR, pyrethroid; SD, standard deviation.
